# Managing multimorbidity: how can the patient experience be improved?

**DOI:** 10.15256/joc.2016.6.75

**Published:** 2016-02-17

**Authors:** Stanimir Hasardzhiev, Luís Mendão, Wolfram Nolte, Bert Aben, Karin Kadenbach

**Affiliations:** ^1^Bulgarian National Patients’ Organization, Sofia, Bulgaria; ^2^Patient Access Partnership, Brussels, Belgium; ^3^Group of Activists on Treatments (GAT), Lisbon, Portugal; ^4^EuropaColon, Germany; ^5^Lynch Syndrome International; ^6^Global Alliance of Mental Illness Advocacy Networks-Europe (GAMIAN-Europe); ^7^Group of the Progressive Alliance of Socialists and Democrats, European Parliament, Brussels, Belgium

**Keywords:** Multimorbidity, multiple chronic conditions, comorbidity, patient experience, Patient Access Partnership

## Abstract

The patient’s experience of their own healthcare is an important aspect of care quality that has been shown to improve clinical and other outcomes. Very little is currently known about patient experience in the management of multimorbidity, although preliminary evidence suggests that it may be poor. Individuals with multimorbidity report better experiences of care when they are knowledgeable and involved in the decision-making, when their care is well coordinated, and communication is good. A greater focus on disease prevention, stronger collaboration between health and social care services, and the provision of more integrated care for people with mental and physical health problems would also help to improve the patient experience. Advocacy groups can amplify the patient voice and improve access to care, as well as provide information and support to patients and their families. Patients have an important role in preventing multimorbidity and improving its management, and should be involved in the development of health policies and the delivery of healthcare services. Inequalities in access to quality healthcare must also be addressed.

## Introduction

There is growing interest amongst researchers, healthcare providers, and policymakers in evaluating the “patient experience” when assessing and attempting to improve the quality of care. Patient experience is a measure of “patient-centredness”, which includes concepts such as respect for patient preferences and values, emotional support, physical comfort, communication and education, continuity and transition, access to care, and coordination of care [1]. Patients place a high priority on patient-centred care, and a good patient experience has been shown to improve clinical and other outcomes [2–7]. 

Very little is known about patient experience in the management of multimorbidity. Studies conducted to date in patients with long-term conditions have tended to focus on single conditions; although one American study found lower ratings of doctor–patient communication amongst people with multiple long-term conditions [8], and a recent British primary care study reported worse experiences in patients with multimorbidity [9]. 

To explore this issue more fully, this article presents the experiences of care reported by three individuals with various multimorbidities, focusing on what went well for them and what could be improved. For these individuals, their own experiences of care have led them towards roles of advocacy – working with patient organizations to represent the views of people with similar conditions, campaigning for change where change is needed, and providing support to other people on similar journeys. This article also describes the role of the European Commission as a patient advocate and presents the first results from a pan-European study evaluating perceptions of access to healthcare by patients and care providers.

## Amplifying the patient voice within Europe

### Karin Kadenbach

As politicians, it is our responsibility to provide as many solutions for as many different groups of people as possible, and when we look at the delivery of health services, we must listen and learn from everyone, including patients themselves. We have all been patients at some point in our lives and have benefitted from the knowledge and skills provided by the healthcare professionals we have encountered. And knowledge is the key. One of the biggest issues we are facing in the European Union (EU) at the moment is how we gather and share the information and knowledge we have built within our member states. Every country in the EU collects outcomes data, but the use of different methodologies and systems precludes us from sharing this information readily and comparing outcomes between countries. One of our most urgent tasks at the European level is to deliver reliable outcomes data (in line with data-protection measures, which include information on the patient experience) to policy- and decision-makers. We need to learn from the best and ensure that this experience and knowledge are shared.

The European Semester, which is the EU’s annual cycle of economic policy guidance and surveillance, has increasingly been focusing on the sustainability of health systems [10]. The centrepiece of the European Semester framework is the publication of its country-specific recommendations (CSR), which set out recommendations for national governments on how to reach the goals of the Europe 2020 Strategy to maintain economic stability and growth [11]. In 2015, 11 countries received a recommendation relating to health, although, perhaps disappointingly, only two related to long-term care or ageing, and none related to the patient experience of care [11]. The European Semester process is a relatively new process that is gradually influencing health systems in the EU and has the potential to enhance the role of patients in helping to shape health policies across the region. To do so, we must ensure that the patient voice is amplified, the patient experience is monitored, and patients are empowered to influence change. 

## How patient power can change clinical practice

### Luís Mendão

Living with multimorbidities resulting from largely preventable conditions (e.g. human immunodeficiency virus [HIV] and hepatitis C virus infections) brings into sharp focus the need to invest in more powerful evidence and rights-based prevention strategies. People with HIV and acquired immune deficiency syndrome (AIDS) played a crucial part in the research advocacy for innovations that have enabled their survival and have increased the safety and tolerability of their treatments. However, they are now living with the long-term consequences of antiretroviral and other therapies, which are associated with the development of metabolic complications and cardiovascular disease. The risk of transmissible diseases, such as hepatitis and HIV, and their long-term health consequences are greatest amongst those with mental health issues and problems with addiction, and typically affect people between the ages of 25 and 45 years. Primary prevention strategies targeted at high-risk groups, such as these, are urgently required and are likely to be cost-effective in the long term. In Portugal, as in many other countries, we see a major imbalance between the investment made in HIV/AIDs prevention and the investment made in treating it, with 85% of the healthcare budget spent on pharmaceuticals, approximately 10–15% on medical care, and only 2% on primary prevention. 

To ease the burden of multimorbidity on the individual and to improve the patient experience, greater collaboration between health and social care providers, with all services offered under one roof, would lessen the treatment burden and help to tackle social exclusion, discrimination, and stigma. More sustainable, regulated, and transparent business models should be developed to address the high cost of pharmaceutical medications and shift the focus from one of “cost” to one of “investment for return”. Incentives should be available to reward good practice and the attainment of good clinical outcomes – penalties should be applied if these outcomes are not achieved.

The European AIDS Treatment Group (EATG) [12], which is close to my heart, stands as an exemplar of how patients can help change clinical practice by participating in clinical research. Working alongside the pharmaceutical industry, and after lobbying hard, the EATG helped to persuade the industry to start including patients with specific comorbidities and multimorbidities in later-stage clinical trials. As a result, many of the new hepatitis C medicines entered the market with sufficient data and indications to be used by patients with multimorbidities, such as those co-infected with HIV and those with blood disorders. Patient power also changed the way that hepatitis C was diagnosed and treated in Portugal, with half of all patients with a diagnosis of hepatitis C now on direct antiviral treatment. Patient organizations, such as the EATG, can help break down the barriers to integrated care for people with multimorbidity by working together across disease boundaries – something that is now being achieved for people with HIV/AIDs and associated conditions.

Good business models are those that are affordable, sustainable, and meet the needs of patients. Patients can, and should, be involved in their own development.

## Involve and empower: tackling multimorbidity for people with mental health problems

### Bert Aben

Bipolar disorder is a severe psychiatric condition that frequently co-exists with long-term physical health problems, such as thyroid disorders, kidney disease, chronic pain, chronic obstructive airways disease, and diabetes [13]. Multimorbidity is common. People with bipolar disorder often live unhealthy lifestyles, which, along with the adverse effects of antipsychotic medications, contribute to high rates of cardiovascular disease and premature death [13]. I have bipolar disorder that I have learnt to live with; however, in the year 2000, I was diagnosed with diabetes and subsequently suffered a heart attack. Fortunately, I live close to a hospital, and survived to tell my story. Fortunately, too, I live in the Netherlands, which, overall, provides good quality care for people with mental health problems, with specialist clinicians – internists – managing both the physical and psychological aspects of the condition. In my experience, problems can arise when different specialists are involved in managing multiple conditions, especially when it comes to medication management and the potential for drug interactions. 

The collective voice of people with mental health problems is relatively quiet, and we often rely on advocates to speak on our behalf. Organizations such as the Global Alliance of Mental Illness Advocacy Networks-Europe (GAMIAN-Europe) [14] offer us a much-needed voice, helping to put patients at the heart of the European healthcare debate. GAMIAN-Europe, which represents approximately 50 European patient organizations, believes that patients can, and should, play a key role in developing positive and proactive policies on mental health issues, and works to provide information and support, facilitate open and inclusive dialogue, and share examples of good practice across the EU. It is my belief that only by involving patients and empowering them to get healthy and to stay healthy will any real progress be made on the issue of multimorbidity. There is much more work still to be done to achieve this goal.

## Improving the patient experience through good care coordination and knowledge

### Wolfram Nolte

A diagnosis of metastatic colon cancer in 2004 changed my life completely. It forced me to face my own mortality, to learn to live without my colon, and to endure punishing rounds of chemotherapy and its long-term side effects. It also forced me to actively engage with patient support groups, to become an expert in my condition, and to recognize the value of patient advocacy during difficult periods of life. Today, I spend much of my time working with advocacy groups associated with my type of cancer in the hope that I can help support other people facing similar issues.

I have been fortunate to have received treatment for my cancer and other comorbidities from medical teams that are well coordinated, discuss and prioritize my healthcare needs, keep me fully informed, and involve me in the decision-making. When my latest round of chemotherapy needed to be paused to allow me to have a much-needed hip replacement, my oncologist and orthopaedic surgeon worked closely together to ensure my risk was minimized and that my chemotherapy could recommence as soon as it was safe to do so. 

My experience as a patient advocate has taught me how to communicate more effectively with healthcare professionals and how to play an active part in my own medical care. Unfortunately, many other patients I have encountered in my work as a patient advocate are less well equipped to navigate their way around complex healthcare systems and many do not receive patient-centred or coordinated care. Health systems are currently not designed to enable patients to contribute to their own healthcare and patient input is rarely sought when health policies are being discussed. 

Patients can play an important role in managing their own multimorbidities, but health systems must be designed to encourage patient advocacy, and patients must be knowledgeable and able to communicate effectively with their healthcare teams. Good care coordination and equipping patients with the knowledge and skills needed to navigate the healthcare system would go a long way towards improving the patient experience when multimorbidity strikes.

## Improving access to quality healthcare in Europe: the Patient Access Partnership

### Stanimir Hasardzhiev

Patients living with multimorbidity need coordinated care that is patient-centred and built around shared decision-making if their needs are to be truly met and their experiences of care improved. Unfortunately, although there is a growing commitment to these principles, the reality for most patients in the EU is that care is fragmented and poorly coordinated. There are also growing inequalities in terms of access to quality healthcare for patients, especially in lower-income countries and those struggling with large budget deficits. 

The Patient Access Partnership (PACT) [15] is a joint initiative led by the Bulgarian National Patients’ Organization [16] and the European European Patients’ Forum Forum [17], and involves multiple stakeholders, including patients, European policymakers, healthcare professionals, academia, and the pharmaceutical industry. The overall vision of PACT is to enable different stakeholders to join forces to develop sustainable solutions to improve access to quality healthcare across Europe. “Access” has been defined by the group as encompassing the five As (5As): availability, adequacy, accessibility, affordability, and appropriateness. 

To try to measure “access” and understand how various stakeholders in Europe perceive access, the PACT has developed a short questionnaire to evaluate different elements of the healthcare system and to identify whether the 5A principles analyse access sufficiently. The questionnaire has been validated in five pilot countries and is being rolled out to all 28 EU countries. The first results available from Belgium, Bulgaria, Cyprus, Estonia, France, and Spain have identified significant differences between the countries in terms of all five aspects of access to healthcare services and between different stakeholders within each country. For example, the availability of primary healthcare services was generally considered to be good by all stakeholders in Belgium, but considered poor by stakeholders in Estonia. In Cyprus, access to long-term care was considered to be adequate by healthcare providers, but inadequate by patient organizations and the pharmaceutical industry. Access to specialist physicians differed markedly between Bulgaria and Belgium, and access to medical devices appeared to be better in France than in Estonia. The affordability of patented medication was considered to be good by most stakeholders in Spain, but poor by the pharmaceutical industry in Belgium. 

Overall, the study has so far confirmed that all five aspects of “access” are relevant and should be evaluated, and that all stakeholders should be examined to gain the fullest picture and to identify all perspectives. The results from all remaining EU countries are currently being evaluated and will be shared soon. However, the preliminary analysis demonstrates that similar conclusions will be drawn from the second wave of answers received.

## Summary and conclusions

Living with multimorbidity poses numerous challenges to the individual, and healthcare systems can help to alleviate or intensify that burden. The patient’s experience of care is an important aspect of care quality and, if the management of multimorbidity is to improve, a better understanding of this concept is required. Patients living with multimorbidity appear to have a better experience of care and better outcomes when their care is well coordinated, they are involved in the decision-making, and communication between all parties is good. Greater investments should be made in preventing multimorbidity and on simplifying care pathways. Patients have an important role in improving the management of multimorbidity, and patients should be involved in the future development of health policies and healthcare services. To make the healthcare systems more accessible, effective, and tailored to the needs of patients with multimorbidity, a multiple stakeholder approach is needed, involving policymakers, healthcare professionals, industry, and the patients themselves.

## References

[1] Gerteis M, Edgman-Levitan S, Daley J, Delbanco TL, Gerteis M, Edgman-Levitan S, Daley J, Delbanco TL (1993). Introduction: medicine and health from the patient’s perspective. Through the patient’s eyes: understanding and promoting patient-centered care.

[2] Sequist TD, Schneider EC, Anastario M, Odigie EG, Marshall R, Rogers WH (2008). Quality monitoring of physicians: linking patients’ experiences of care to clinical quality and outcomes. J Gen Intern Med.

[3] Greenfield S, Kaplan S, Ware JE (1985). Expanding patient involvement in care. Effects on patient outcomes. Ann Intern Med.

[4] Greenfield S, Kaplan SH, Ware JE, Yano EM, Frank HJ (1988). Patients’ participation in medical care: effects on blood sugar control and quality of life in diabetes. J Gen Intern Med.

[5] Stewart MA (1995). Effective physician-patient communication and health outcomes: a review. Can Med Assoc J.

[6] Fremont AM, Cleary PD, Hargraves JL, Rowe RM, Jacobson NB, Ayanian JZ (2001). Patient-centered processes of care and long-term outcomes of myocardial infarction. J Gen Intern Med.

[7] Beach MC, Keruly J, Moore RD (2006). Is the quality of the patient-provider relationship associated with better adherence and health outcomes for patients with HIV?. J Gen Intern Med.

[8] Fung CH, Setodji CM, Kung FY, Keesey J, Asch SM, Adams J (2008). The relationship between multimorbidity and patients’ ratings of communication. J Gen Intern Med.

[9] Paddison CA, Saunders CL, Abel GA, Payne RA, Campbell JL, Roland M (2015). Why do patients with multimorbidity in England report worse experiences in primary care: Evidence from the General Practice Patient Survey. BMJ Open.

[10] Azzopardi-Muscat N, Clemens T, Stoner D, Brand H (2015). EU Country Specific Recommendations for health systems in the European Semester process: trends, discourse and predictors. Health Policy.

[11] European Commission (2015). European Semester: country-specific recommendations.

[12] European AIDS Treatment Group http://www.eatg.org.

[13] Smith DJ, Martin D, McLean G, Langan J, Guthrie B, Mercer SW (2013). Multimorbidity in bipolar disorder and undertreatment of cardiovascular disease: a cross sectional study. BMC Med.

[14] Global Alliance of Mental Illness Advocacy Networks-Europe (GAMIAN-Europe) http://gamian.eu.

[15] Patient Access Partnership (PACT) http://www.eupatientaccess.eu.

[16] Bulgarian National Patients’ Organization http://www.npo.bg.

[17] European Patients Forum http://www.eu-patient.eu.

